# Protocol for the safety and efficacy of fecal microbiota transplantation liquid in children with autism spectrum disorder: a randomized controlled study

**DOI:** 10.3389/fmicb.2023.1236904

**Published:** 2023-08-22

**Authors:** Jinying Wei, Jiayi Chen, Xiaohui Fang, Tianyu Liu, Yanhan Yuan, Jinping Zhang

**Affiliations:** ^1^College of Food Science and Technology, Shanghai Ocean University, Shanghai, China; ^2^Shanghai Sixth People’s Hospital Affiliated to Shanghai Jiao Tong University School of Medicine, Shanghai, China; ^3^Pediatrics, Shanghai Sixth People’s Hospital, Shanghai, China

**Keywords:** Autism Spectrum Disorder, fecal microbiota transplantation, gut microbiota, safety, efficacy, protocol ChiCTR2200058459, pre-results

## Abstract

**Background:**

Autism Spectrum Disorder (ASD) is a neurodevelopmental disorder characterized by deficits in social interaction, repetitive behavior and language impairment, and its worldwide prevalence has been found to be increasing annually in recent years. Till now, ASD is uncurable as its pathogenesis remains unknown. However, studies on both animals and humans have demonstrated that fecal microbiota transplantation (FMT) may ameliorate the symptoms of ASD, as well as gastrointestinal symptoms. Nonetheless, there is still no agreement regarding the optimal dosage or duration of FMT treatment for individuals with ASD.

**Methods:**

This clinical study is a double-blind, randomized, interventional trial conducted at a single center. The aim is to investigate the safety and efficacy of a pediatric formulation of FMT for ASD. A total of 42 children between the ages of 3–9 with ASD will be randomly assigned in a 2:1 ratio to either an FMT treatment group (*n* = 28) or a placebo group (*n* = 14), forming cohort 1. Additionally, 30 healthy children of similar age and gender will be recruited as the control group (cohort 2). Cohort 1 will be assessed using a variety of scales, including the Autism Behavior Checklist, Childhood Autism Rating Scale, Social Responsiveness Scale, Gastrointestinal Symptom Rating Scale, Children’s Sleep Habits Questionnaire, and Psychoeducational Profile (Third Edition). These assessments will evaluate the effectiveness of FMT in reducing core symptoms and comorbidities (such as gastrointestinal symptoms and sleep disturbances) in children with ASD. The study will use metagenomic and metabolomic sequencing to assess changes in the composition and structure of the intestinal flora and its metabolites in blood, urine, and feces following treatment. Furthermore, the study will evaluate the acceptability of the FMT formulation by participants’ legal guardians and investigate differences in the intestinal flora and metabolism in the FMT group before and after treatment compared to 30 healthy children.

**Clinical trial registration:**

https://www.chictr.org.cn/, identifier ChiCTR2200058459.

## 1. Introduction

Autism Spectrum Disorder (ASD)^1^ is a complex neurodevelopmental disorder characterized by core features such as social deficits, stereotyped behaviors, and language impairments (Coretti et al., 2018). Over the past 20 years, there has been a significant rise in the prevalence of ASD, with the current rate in the US estimated to be 1 in 44 children according to the 2021 CDC report, posing a significant challenge to both public health and families. In addition to genetic factors, gut microbes and their metabolites have become a popular area of research in recent years (Qi et al., 2021). The human gut contains approximately 40 trillion bacteria, which is 5–10 times more than the total number of cells in the body. This microbial population also has approximately 150 times more genes than the human body, often called the “second brain” of the body (Sun et al., 2019). It is reported that people with ASD often suffer from a combination of gastrointestinal symptoms such as constipation and diarrhea, as well as altered gut microbiology, mainly in the form of abnormal bacteria and metabolites (Rose et al., 2018). For individuals with ASD, gastrointestinal symptoms aggravate their sleep problems, and conversely inadequate sleep quality and quantity also aggravate their ASD symptoms (Cortese et al., 2020). In addition, children with ASD who suffer from sleep disorders also suffer from imbalances in their gut flora (Han et al., 2022).

The gut microbiota is highly specific, which can lead to varying conclusions among different studies regarding the gut microbiota characteristics in individuals with ASD. Research has shown that compared to healthy individuals, children with ASD tend to have a deficiency of beneficial gut bacteria. Further, there is higher level of Bacteroidetes, Firmicutes, and Actinobacteria at the phylum level in children with ASD. At the genus level, children with ASD tend to have higher level of Prevotella, Faecali bacterium and Bacteroides while having lower levels of Akkermansia and *Bifidobacterium longum* (Ristori et al., 2019; Iglesias-Vazquez et al., 2020; Zou et al., 2020). Patients with both severe ASD and high Bacteroidetes have significantly higher total short-chain fatty acids (Qi et al., 2021), and in pregnant rats, it was found that abnormalities in these total short-chain fatty acids could lead to ASD-like behavior in their offspring (Alharthi et al., 2022). Patients with ASD exhibit abnormal levels of plasma tryptophan and glutamate and their respective metabolites. Also, both tryptophan and glutamate, along with their metabolites, have significant impacts on brain development (Montanari et al., 2022). Microorganisms in the gastrointestinal tract play a crucial role in regulating amino acid digestion, absorption, synthesis and metabolism and also have an impact on the central nervous system of the host (Lin et al., 2017). Tryptophan plays an important role in the pathogenesis of ASD (Wang et al., 2020). Changes in gut microbiota can impact tryptophan metabolism, and the administration of probiotics can facilitate the conversion of tryptophan to serotonin, also known as 5-hydroxytryptamine (Kong et al., 2022). Short-chain fatty acids found in the gut can down-regulate the expression of the Neurexins gene and increase the production of catecholamines, ultimately leading to improved ASD symptoms in rats (Nankova et al., 2014). Gut microbes produce signaling molecules that promote the synthesis of neurotransmitters such as serotonin, dopamine, and gamma-aminobutyric acid. Furthermore, studies have shown that *Bifidobacterium longum* can improve neuroinflammation resulting from tryptophan abnormalities in rats with Autism Spectrum Disorder (ASD) (Liu et al., 2021). The bidirectional communication between gut microbes and the central nervous system forms a “microbe-gut-brain axis (Bonaz et al., 2018), which may regulate neurodevelopment and neurodegenerative diseases, including Autism Spectrum Disorder, anxiety and depression, Alzheimer’s disease, and Parkinson’s disease, through metabolic, vagal, and immune modalities (Fattorusso et al., 2019; Ansari et al., 2020). Although the mechanism linking gut microbes to the pathogenesis of ASD remains unknown, studies have shown that mice transplanted with gut flora from children with autism develop autistic symptoms (Sharon et al., 2019). Therefore, bacterial and metabolic markers may play a significant role in the pathogenesis of ASD, but further research is necessary to identify these markers.

Fecal microbiota transplantation (FMT) is a procedure that involves transferring fecal microorganisms from a healthy individual to a patient’s gastrointestinal tract, with the aim of improving the intestinal microecology and treating the disease (Liu et al., 2022). Studies have shown that FMT can improve symptoms of ASD, as well as gastrointestinal symptoms and intestinal microecology (Tan et al., 2021). The intestinal microecology is diverse and complex, and FMT contains around 1,000–1,150 functional bacteria that colonize the host gut more efficiently than prebiotics or probiotics (Vendrik et al., 2020). Although no serious adverse events have been identified in studies of FMT for ASD, clinical adverse effects such as fever and allergies have been observed in children with ASD treated with FMT (Zhao et al., 2019). Therefore, the efficacy and safety of FMT need to be further evaluated. More research is needed to understand how FMT improves ASD symptoms by altering intestinal flora and metabolites (Xu et al., 2021). Additionally, there is still no consensus on the optimal dose or duration of FMT for the treatment of ASD. This study is a double-blind randomized controlled trial with the primary aim of assessing the improvement of ASD symptoms with FMT. By analyzing the correlation between changes in the scale of autism-related symptoms and gut flora and metabolites in a reasonable grouping, sample size, dosing method and treatment phase, we sought to identify the unique bacterial and metabolic markers of ASD that may provide additional evidence for the “microbial-gut-brain axis” and the treatment of ASD symptoms with FMT. This study will have important implications for treating patients with ASD associated with gut microecological dysbiosis.

**Figure 1:**
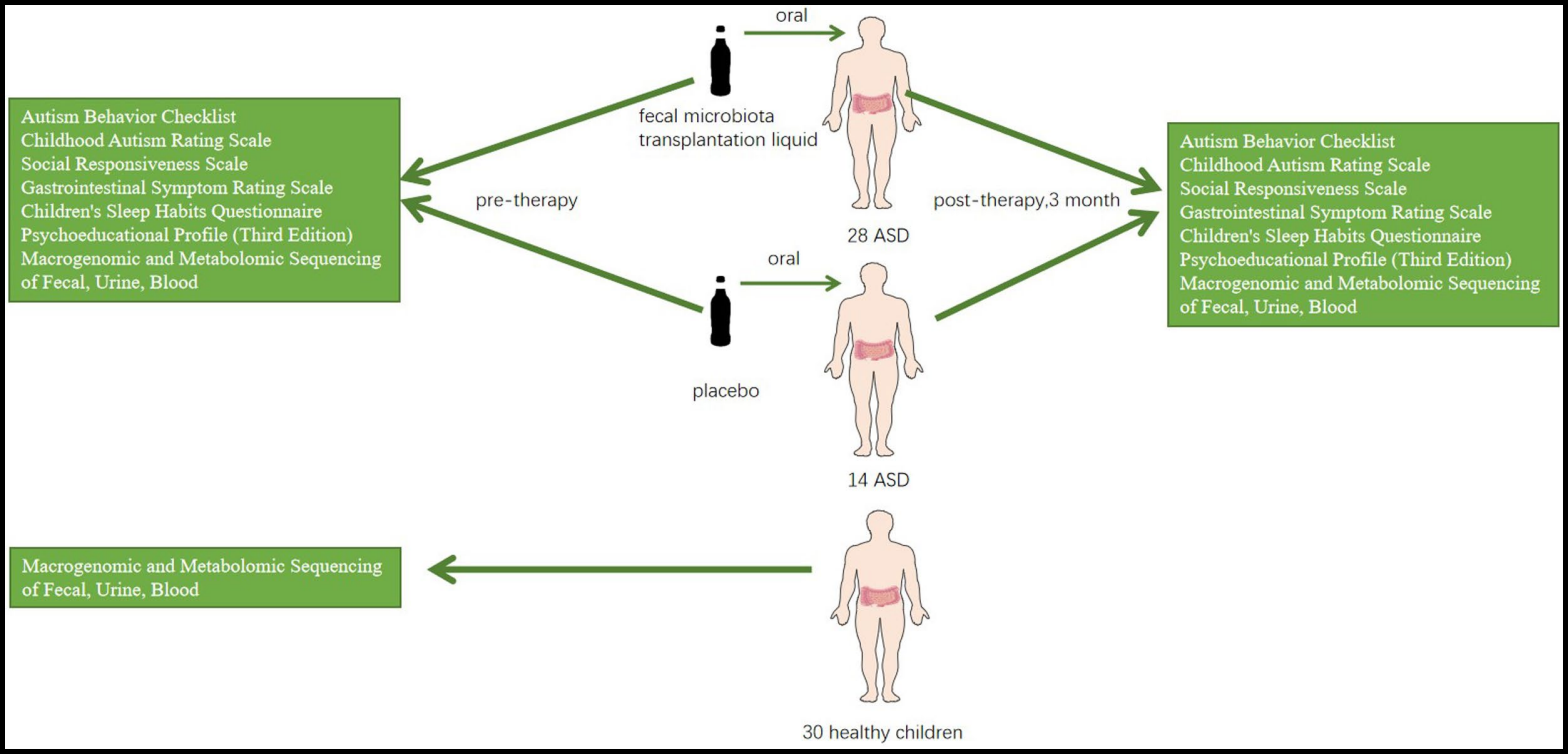
An experimental FMT treatment group (*n* = 28) and a placebo group (*n* = 14) are set up, with healthy children (*N* = 30) as controls. A series of scales and metagenomic and metabolomic sequencing of blood, urine, and feces will be used to compare the results of the three groups.

This is a double-blind randomized controlled trial aimed at assessing the effectiveness of FMT in improving symptoms of ASD (Figure 1). The main objective of this study is to evaluate the effectiveness of the FMT technique in improving symptoms in children with ASD and to identify the core symptoms of ASD that can be improved by FMT. Additionally, there are four secondary aims: (1) to compare changes in the composition of gut flora in children with ASD before and after FMT, (2) to compare differences in gut flora between children with ASD and healthy children, (3) to assess the safety of the FMT pediatric formulation in children with ASD, and (4) to evaluate the acceptability of the FMT pediatric formulation to children with ASD and their legal guardians. The exploratory aim of the study is to identify changes in the metabolomics of children with ASD before and after receiving FMT.

## 2. Methods and analysis

### 2.1. Study design

This is a single-center, double-blind, double-modeled, randomized, interventional clinical study aiming at exploring the efficacy and safety of FMT formulation on an interventional treatment of children with ASD. The degree of improvement in their symptoms will be assessed using several scales. Additionally, changes in the composition and structure of their intestinal flora and metabolites of the flora in their blood, urine and feces before and after treatment will also be assessed by macro-genomic sequencing and metabolomics. The study will also evaluate the differences in the gut flora of children with ASD and healthy children and the acceptability of FMT pediatric preparations by children with ASD and their legal guardians.

The primary endpoint of the study is the change from baseline in Childhood Autism Rating Scale (CARS) scores in children with ASD after 12 and 24 weeks of treatment. The secondary endpoints are the variations in assessment scales, and gut flora and included five main points:

(1) Variations in scores from baseline in the Social Responsiveness Scale (SRS), Autism Behavior Checklist (ABC), Psychoeducational Profile Third Edition (PEP3), Gastrointestinal Symptom Rating Scale (GSRS), and Children’s Sleep Habits Questionnaire (CSHQ) after 12 and 24 weeks of treatment;
(2) Variations in gut flora characteristics (including alpha-diversity, flora composition, beta-diversity, colonizing bacteria, differential bacteria, and metabolic pathway function) in children with ASD after treatment compared to baseline and donors;
(3) Differences in intestinal flora characteristics (including alpha-diversity, flora composition, beta-diversity, differential bacteria, and metabolic pathway function) between children with ASD (baseline) and healthy children;
(4) The incidence of adverse events during the treatment of children with ASD;
(5) Variations in the FMT acceptance scale scores for participants with ASD and their legal guardians.

The patients will be divided into two cohorts: Cohort 1 will include 42 children aged 3–9 years diagnosed with ASD, while Cohort 2 will include 30 healthy children aged 3–9 years. The sex ratio and age range of children enrolled in Cohort 1 and Cohort 2 will be kept roughly the same. In Cohort 1, subjects who meet the entry criteria will undergo a two-week screening visit after the legal guardian signs the informed consent. They will then be randomly allocated to either the trial or control group in a 2:1 ratio. Children in the trial group will undergo rehabilitation and FMT, while the control group will receive rehabilitation and placebo. All children will undergo a 12-week treatment period following the completion of bowel preparation and followed up for 12 weeks, during which they will receive only rehabilitation. A homogenous rehabilitation scheme will be performed on all the ASD children in Cohort 1. The study participants will be discharged at week 25 after the end visit. In Cohort 2, after the legal guardian signs the informed consent, they will undergo a physical examination, assessment of vital signs, and various laboratory examinations within two weeks to confirm their health status. Blood, urine, and fecal samples will be collected within one week, and the children will be discharged. Metabolomics analysis will be performed on blood and urine samples, while macro-genomic sequencing and metabolomics analysis will be conducted on fecal samples.

### 2.2. Inclusion/exclusion criteria

The study participants in each cohort will meet all inclusion criteria to be eligible for this trial. For cohort 1, the patients will:

(1) have a diagnosis of autism as outlined by a child psychiatrist according to the Diagnostic and Statistical Manual of Mental Disorders (5th edition) and positive results on both the Autism Diagnostic Observation Schedule and Autism Diagnostic Interview-Revised assessments;
(2) age 3–9 years, regardless of gender;
(3) not have immunodeficiency disorders;
(4) provide signed ICF by participants’ legal guardians who fully understand the study;
(5) have an ABC score ≥ 62 at the time of screening;
(6) meet two of the following scale scores at the time of screening: Childhood Autism Rating Scale (CARS) score ≥ 38; Social Responsiveness Scale (SRS) score ≥ 75.

For cohort 2, the patients will:

(1) age 3–9 years;
(2) have a male to female ratio as close to cohort 1 as possible;
(3) provide signed ICF by participants’ legal guardians who fully understand the study;
(4) have normal physical examination, vital signs and important laboratory test results.

Subjects meeting any of the following exclusion criteria will not be allowed to participate.

(1) Use of probiotics, antibiotics, antifungal, prebiotic and other drugs affecting the intestinal microflora in a planned way within the last 3 months or in the following 6 months.
(2) Diagnosis of intestinal obstruction (including partial obstruction), or severe ulcerative damage to the bowel;
(3) Diagnosis of an immunodeficiency disorder or autoimmune disease, including but not limited to rheumatoid arthritis, systemic lupus erythematosus, ankylosing spondylitis, desiccation syndrome, Hashimoto’s thyroiditis, toxic diffuse goiter, autoimmune hemolytic anemia, idiopathic thrombocytopenic purpura, Crohn’s disease, psoriasis, glomerulonephritis, nephrotic syndrome;
(4) A previous history of severe food or drug allergy;
(5) A history of severe fever and/or severe infection within 7 days prior to enrolment;
(6) Renal insufficiency and hepatic dysfunction, for instance, alanine aminotransferase level > 1.5 times the normal upper limits, aspartate aminotransferase level > 1.5 times the normal upper limits, or serum creatinine level > 1.5 times the normal upper limits;
(7) Be in an immunosuppressed state (i.e., presence of a neoplastic disease or organ transplantation) underwent or undergoing chemotherapy;
(8) Diagnosed with inflammatory bowel disease, celiac disease, irritable bowel syndrome or eosinophilia, oesophagitis, eosinophilic gastroenteritis or similar conditions;
(9) Diagnosis of blindness, deafness, cerebral palsy, etc.;
(10) Participation in another clinical study or an interventional clinical study with non-conventional rehabilitation within 4 weeks prior to enrolment;
(11) A previous history of HIV, hepatitis B virus, hepatitis C virus or syphilis infection, or a parent with HIV infection;
(12) A treatment of intra-abdominal surgery (excluding appendectomy or cholecystectomy) within the past 60 days prior to screening and/or planned invasive surgery/hospitalization during the study period;
(13) Presence of certain prominent symptoms, including but not limited to stereotyped repetition, aggression, self-injury, vandalism, severe emotional behavioral problems and extreme hyperactivity, and requiring pharmacological treatment as per the principal investigator assessment;
(14) Poor compliance (i.e., therapeutic drinks taken at less than 80%), using the following formulas to estimate:

- Dose = total dose - remaining dose;
- Adherence rate (a quantitative indicator of adherence) = (dose taken/total amount) x 100%.

### 2.3. Sample size estimation

For Cohort 1, a total of 42 subjects will be randomized in a 2:1 ratio (28 in the trial arm: 14 in the control arm) to provide sufficient statistical power for determining the effectiveness of FMT in improving CARS scores compared to placebo. It is anticipated that, after 25 weeks of treatment, the mean reduction in CARS scores for the FMT group will be 5 points. Additionally, using a one-sided alpha level of 5 and 80% power, the proposed sample size will be able to detect the expected differences between the two groups. Considering a 15% dropout rate, the final sample size for Cohort 1 is expected to be 42 cases.

For Cohort 2, a sample size of 30 cases is considered sufficient to compare gut flora differences between children with ASD and healthy children.

### 2.4. Randomization and blinding

The Interactive Web Response System (IWRS) will be used to randomize subjects in Cohort 1. Prior to enrollment, a random coding table will be generated by non-blinded statisticians using the SAS 9.4 randomization process and imported into IWRS. The IWRS will be accessed by investigators using their respective codes, and eligible subjects will be assigned a unique drug number in IWRS and will receive the study drug corresponding to the drug number. Subjects who have been randomized, regardless of whether they are receiving study medication or not, cannot be reassigned to other subjects if the study is terminated for any reason.

Cohort 1 will be performed in a double-blind manner. From the time of randomization, their treatment will be blinded, as well as the investigator, study team and anyone involved in the trial until the database is locked. The study drugs will also be blinded by non-blinded personnel not involved in this study, according to a drug randomization coding list provided by non-blinded statisticians. The computer program generating the randomization codes and the randomization code sheet will be used as a blinded base and kept securely by the unblinded team. The blinding will be kept strictly confidential until the final blinding, except in the circumstances such as emergency unblinding or suspected unexpected serious adverse reactions, in which case individual blinding may be performed by designated project personnel. Blinding personnel involved in the execution of the trial will not be informed of any of the subject’s blinding status until final blinding is completed, except in cases of emergency blinding.

### 2.5. Interventions

#### 2.5.1. Test drug

The drug in this study will be XBI-061, and the placebo will be XBI-061 placebo, both having the same appearance, smell, weight and available in two sizes: 6 mL/bottle and 0.6 mL/bottle, containing 1 × 10^10^ to 1 × 10^11^ live bacteria and 1 × 10^9^ to 1 × 10^10^ bacteria, respectively. The ASD children in the placebo group will receive the same dose every time as the treatment group. One bottle of the bacterial liquid or placebo will correspond to approximately 70 mL of the therapeutic drink. Based on the volume the child can tolerate, the bacterial liquid or placebo will be removed from the cold refrigerator, dissolved in a therapeutic drink, shaken well, and immediately consumed.

#### 2.5.2. Experimental flow

##### 2.5.2.1. Cohort 1

**Figure 2:**
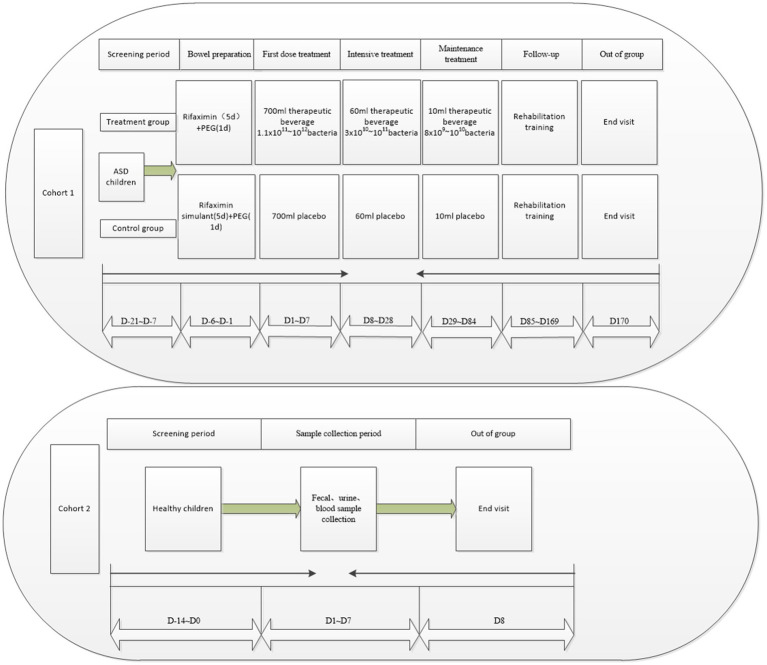
Overall process of the clinical trial.

The study flow for Cohort 1 will be divided into seven phases: screening period, bowel preparation, first treatment dose, intensive treatment, maintenance treatment, follow-up, and exit (Figure 2). The patients will undergo stool and urine sample collection, relevant examinations, and scale assessments (Table 1). During the screening period (D-21 ~ D-7), the eligible ASD patients will be assessed based on the implemented scales and complete all required examinations, and the baseline data will be recorded. In the bowel preparation phase (D-6 ~ D-1), patients in both the trial and control groups will undergo bowel preparation with oral rifaximin suspension or rifaximin mimetic for five days at 0.1 g each time, four times daily, followed by one day of polyethylene glycol bowel cleansing. The first treatment dose (D1 ~ D7) will involve administering a total of approximately 700 mL of the therapeutic drink (containing 66 mL of bacterial liquid or placebo) orally over 3 to 5 consecutive days of the week, depending on the subject’s acceptance of the therapeutic drink and the volume available for each dose. Intensive treatment (D8 ~ D28) will involve an intensive dose of approximately 60 mL of the treatment drink (containing 6 mL of bacterial fluid or placebo) once a week for 3 weeks. During the maintenance treatment phase (D29 ~ D84), once weekly doses of approximately 10 mL of the treatment drink (containing 0.6 mL of bacterial liquid or placebo) will be administered for 8 weeks. During the follow-up period (D85 ~ D169), the subjects will receive the same rehabilitation as during the treatment period. They will be discharged at week 25 after completion of the end-visit examination (D170). Throughout the treatment period, they will be asked to adhere to the “Dietary Manual for People with Autism Receiving FMT Therapy” as closely as possible, with a legal guardian completing a weekly dietary record form.

**Table 1:** Inspection schedule for cohort 1.

Parameters	Baseline	Bowel preparation	Treatment period						Follow-up
			First dose treatment	Intensive treatment	Maintenance treatment				
Time window	D-21 ~ D-7	D-6 ~ D-1	D1 ~ D7	D8 ~ D28	D29 ~ D42	D43 ~ D56	D57 ~ D71	D71 ~ D84	D85 ~ D169
Informed consent	X								
Inclusion and exclusion criteria	X								
Demographic data^a^	X								
Medical and treatment history	X								
Height	X								
Weight	X							X	X
Vital signs^b^	X							X	X
Allergy history	X								
Health checkup	X							X	X
Routine blood test	X							X	X
Blood biochemistry	X							X	X
Urinalysis	X							X	X
Fecal routine	X							X	X
Fecal sample collection^c^	X			X		X		X	X
Urine sample collection^c^	X			X		X		X	X
Blood sample collection^c^	X							X	X
Bowel preparation		X							
FMT			X						
CARS	X			X		X		X	X
SRS	X			X		X		X	X
GSRS	X			X		X		X	X
ABC	X			X		X		X	X
CSHQ	X			X		X		X	X
PEP3	X			X		X		X	X
rehabilitation training	X								
Acceptance survey^d^			X	X				X	
Diet Record	X								
Adverse event record	X								
Combined medication record	X								

FMT, Fecal Microbiota Transplantation; CARS, Childhood Autism Rating Scale; GSRS, Gastrointestinal Symptom Rating Scale; ABC, Autism Behavior Checklist; PEP3, Psychoeducational Profile(Third Edition); SRS, Social Responsiveness Scale; CSHQ, Children’s Sleep Habits Questionnaire.

1 Demographics: date of birth, age, sex, ethnicity.

2 Vital signs: temperature, blood pressure, heart rate (pulse), respiratory rate.

3 Sample collection:

a Stool sample collection: Stool samples will be collected for macro-genome sequencing and metabolomic analysis during the screening period, every fortnight after the start of treatment for macro-genome sequencing, and every four weeks for metabolomic analysis until the end of the study.

b Urine sample collection: Urine samples will be collected for metabolomic analysis during the screening period and every four weeks from the start of treatment until the end of the study.

c Blood sample collection: Blood samples will be collected for metabolomic analysis during the screening period, at the end of treatment (week 13), and at the end of the study (week 25) for metabolomic analysis and safety data collection in the blood.

d Acceptance questionnaires: Part A and Part B questionnaires will be completed by legal guardians only during the screening period; Part A and Part B questionnaires will be received by subjects and/or legal guardian at the end of the first dose of treatment, booster treatment and maintenance treatment, respectively, (see appendix for details).

##### 2.5.2.2. Cohort 2

After the legal guardian signs the ICF, during the screening period (D-14 to D0), the patient’s demographic information (including date of birth, age, sex, and ethnicity), medical and treatment history, allergy history, vital signs (such as temperature, blood pressure, heart rate, and respiratory rate), physical examination, height and weight measurements, as well as data from routine blood, blood biochemistry, urine and fecal tests will be collected and evaluated to determine the patient’s health status.

After the legal guardian signs the ICF, the screening period (D-14 to D0) will involve collecting demographic data (such as birth date, age, gender, and nationality), important medical history, treatment history, and allergy history of the subjects. Vital signs, including temperature, blood pressure, heart rate, respiratory rate, physical examination, height and weight measurements, as well as data from routine blood, blood biochemistry, urine and fecal tests, will also be recorded to determine the patient’s health status.

During the sampling period (D1 to D7), one stool sample will be collected for macroeconomic and metabolomic analysis, and one urine sample and a blood sample will also be collected for metabolomic analysis (Table 2 and Figure 2), following which they will be eligible for discharge.

**Table 2:** Inspection schedule for Cohort 2.

Parameters	Baseline	Sample collection period
Time windows	D-14 to D0	D1 to D7
Informed consent	X	
Inclusion and exclusion criteria	X	
demographic data^1^	X	
Medical and treatment history	X	
Vital signs^2^	X	
allergy history	X	
health checkup	X	
routine blood test	X	
Blood biochemistry	X	
Urinalysis	X	
Fecal routine	X	
Fecal sample collection^3^		X
Urine sample collection^3^		X
Blood sample collection^3^		X

1 Demographics: date of birth, age, sex, ethnicity.

2 Vital signs: temperature, blood pressure, heart rate (pulse), respiratory rate.

3 Stool sample collection: Stool samples will be collected for macro-genome sequencing and metabolomic analysis during the screening period.

3 Urine sample collection: Urine samples will be collected for metabolomic analysis during the screening period.

3 Blood sample collection: Blood samples will be collected for metabolomic analysis during the screening period.

### 2.6. Statistical analysis

Bioinformatics and statistical analyses will be conducted by professionals using specialized software tools. Analysis of clinical scale results will involve comparing changes in ABC, CARS, SRS, GSRS, CSHQ and PEP3 scales before and after FMT. Differences in scale results between pre- and post-treatment and control groups will also be assessed. To analyze gut flora and metabolites, we will examine alpha-diversity, beta-diversity, bacterial symbiotic networks, and metabolome analysis using the R language MetaboAnalystR package. This package will enable the quantification of metabolite content and the identification of characteristic metabolites. Mann–Whitney U and Kruskal-Wallis tests will be used to analyze differences in gut flora and metabolites between groups. To explore the correlation between gut flora, metabolites, and improvements in ASD symptoms, gastrointestinal symptoms, and sleep symptoms, we will use Spearman’s correlation test.

## 3. Risk prevention and management

A randomized, double-blind study with *N* = 52 patients suffering from irritable bowel syndrome evaluated the safety and efficacy of oral FMT capsules. It revealed that diarrhea was the only statistically significant adverse event, and no serious adverse events (SAEs) were observed (Halkjaer et al., 2018). Other FMT-related studies found that nausea and diarrhea were the most common adverse events associated with oral FMT capsules. However, these symptoms are generally self-alleviating and do not require treatment. When completing the adverse event form, all relevant information will be collected, including the adverse event and associated symptoms, time of occurrence, severity, duration, correlation with the bacterial liquid/placebo, action taken, and final outcome. The supervisor will confirm that the adverse events are reported within the specified period in accordance with relevant laws and regulations, the trial protocol, ethics committee, and sponsor’s requirements. In the event of an adverse event, the investigator will closely observe the subject. In the case of SAEs, subsequent FMT will be immediately discontinued.

1. If a severe allergic reaction occurs, standard anti-allergy measures will be taken, and medication will be administered if there are no contraindications.
2. If an inflammatory bowel disease attack occurs, standard treatment measures for inflammatory bowel disease attacks should be taken, including stool and blood sample collection. Traceability testing and analysis should be initiated immediately to determine the cause of the attack.
3. In the event of a severe infection, blood cultures and stool tests will be performed immediately to identify the causative organism and the most effective antimicrobial agent. Broad-spectrum antibacterial drugs and supportive therapy will be administered while awaiting test results, which may help to boost the patient’s resistance.
4. If a serious infection or any other emergency related to the FMT intervention occurs during the trial, immediate action will be taken. Blood and stool samples will be collected from the subject for pathogen detection and analysis to trace the source of the causative pathogen before administering any antimicrobial agent

## 4. Ethics and dissemination

This study has been approved by the Ethics Committee of Shanghai Sixth People’s Hospital in December 2021 and registered with the China Clinical Trials Center (registration number: ChiCTR2200058459). The investigator will explain the study’s background, pharmacological characteristics of the study drug, trial protocol, and potential benefits and risks of participating in the trial to each subject’s legal guardian and obtain written informed consent before enrolling the subject. Good Clinical Practice and International Council for Harmonization of Technical Requirements for Pharmaceuticals for Human Use guidelines will be followed by the investigator and all participants. A dedicated team, independent of the Clinical Research Center project team, will manage and monitor the study. Participating subjects will be legally covered by insurance. The study’s results will be published in medical journals or conferences, while the subjects’ information will be kept confidential as required by law. The subjects’ personal information will not be disclosed except as mandated by relevant laws. Patient information will be accessed by government authorities and hospital ethics committees when required.

## 5. Discussion

ASD is a complex and heterogeneous disease with multiple phenotypes. Its underlying cause is unknown, and no curative drugs or treatments are currently available. However, increasing evidence from animal studies and clinical research supported a link between the microbe-gut-brain axis and ASD (Cryan et al., 2019; Martinez-Gonzalez and Andreo-Martinez, 2020). Gastrointestinal symptoms are a common complication in children with ASD, with affected children four times more likely to experience symptoms such as diarrhea, constipation, and abdominal pain than non-ASD children (Patusco and Ziegler, 2018). Some of these symptoms, such as reflux and vomiting, can disrupt the daily activities of children with ASD and are often accompanied by other symptoms, such as an imbalanced intestinal flora, increased intestinal permeability, inflammation, and food allergies (Buie et al., 2010).

Furthermore, there were evidences suggesting that FMT may be effective in improving both ASD behavior and gastrointestinal symptoms by improving intestinal microecology. Preclinical experiments have shown that FMT could improve autistic behavior, and fecal bacteria from wild mice have been found to improve autistic behavior, metabolic levels, memory, and social skills in ASD mice (Li et al., 2020). Kang et al. used FMT in combination with antibiotics, omeprazole and Movi Prep therapeutic drinks, which increased the abundance of Bifidobacterium, Prevotella, and Desulfovibrio, leading to improvements in ASD and gastrointestinal symptoms that lasted for at least 8 weeks (Kang et al., 2019) which also led to changes in serum and intestinal metabolites (Kang et al., 2020). Li et al. found that FMT improved gastrointestinal symptoms and ASD symptoms, promoted a shift in the intestinal flora of children with ASD to that of typical neurological children, and significantly altered serum levels of neurotransmitters (Li et al., 2021). Additionally, Zhao et al. found that FMT was statistically different in improving gastrointestinal symptoms and ASD symptoms, improved gut microbiology, and caused only mild, transient adverse events in 29.2% of patients (Zhao et al., 2019). These studies demonstrate that FMT is a safe and effective treatment that contains at least 1,000–1,150 functional bacteria (Zhang et al., 2012), making it an ideal treatment option for improving gut microecology and the effectiveness of treatment for ASD.

Although FMT has been shown to effectively improve the symptoms of ASD, there is still no consensus on the optimal dose and treatment regimen for FMT in ASD patients (De Angelis et al., 2015). Chen et al. used an individualized daily intake of fecal bacteria (Chen et al., 2022). Our study will use standard bacterial fluids administered in three phases: first-dose treatment, intensive treatment, and maintenance treatment. Prior to treatment, antibiotics were used to clean the bowel, making this the first study to use bacterial fluids in three phases of treatment, which has significant implications for the further development of clinical applications of FMT. The critical period after antibiotic use was likely the most favorable for colonizing the gut by foreign bacteria (Kang et al., 2019). Therefore, our study’s first week of treatment will involve a high bacterial dose, followed by three weeks of intensive treatment and eight weeks of maintenance treatment. The bacterial liquid in this study will be dissolved in drinks, making it easier for children with ASD to swallow, which will increase the acceptability of FMT treatment. This study hypothesizes that FMT will target specific ASD symptoms, gastrointestinal symptoms and sleep symptoms, and aim to identify the bacteria and metabolites associated with improved symptoms. This study is a double-blind randomized controlled trial, but some limitations still exist. Firstly, the sample size might be small, with only 42 patients enrolled in this study. Further expansion of sample size will need to demonstrate the efficacy of fecal microbiota transplantation fluid for autism. Secondly, future trials will group children with ASD according to different core symptoms to explore the precise targeting of FMT treatment for ASD symptoms and compare the effects of FMT treatment within and between groups. Thirdly, a longer follow-up time will enable better observation of the duration of improvement in ASD symptoms and the safety and efficacy of FMT. This study will have only one follow-up and willnot track the long-term impact of FMT on children with ASD. Fourthly, besides diet, probiotics and other factors, there are many factors affecting gut microbiota. This study will not fully consider the mixed factors affecting gut microbiota, such as living habits, age, etc.

## Data availability statement

The raw data supporting the conclusions of this article will be made available by the authors, without undue reservation.

## Ethics statement

The studies involving humans were approved by the Ethics Committee of Shanghai Sixth People’s Hospital. The studies were conducted in accordance with the local legislation and institutional requirements. Written informed consent for participation in this study was provided by the participants’ legal guardians/next of kin.

## Author contributions

JZ is responsible for the experimental design and leading the experiment. JW is responsible for writing the manuscript. JC is responsible for revising the manuscript and XF, TL, and YY is responsible for obtaining the experimental data. All authors contributed to the article and approved the submitted version.

## Funding

The main source of funding for this study was the “Clinical Study on the Efficacy and Safety of Gut Flora Transplantation Technology for Children with Autism Spectrum Disorders (XCT0273003)”.

## Conflict of interest

The authors declare that the research was conducted in the absence of any commercial or financial relationships that could be construed as a potential conflict of interest.

## Publisher’s note

All claims expressed in this article are solely those of the authors and do not necessarily represent those of their affiliated organizations, or those of the publisher, the editors and the reviewers. Any product that may be evaluated in this article, or claim that may be made by its manufacturer, is not guaranteed or endorsed by the publisher.

## Footnotes

^1^ https://www.cdc.gov/ncbddd/autism/articles.html

## References

[ref1] Alharthi A. Alhazmi S. Alburae N. Bahieldin A. (2022). The human gut microbiome as a potential factor in autism Spectrum disorder. Int. J. Mol. Sci. 23:23031363. doi: 10.3390/ijms23031363, PMID: 35163286 PMC8835713

[ref2] Ansari F. Pourjafar H. Tabrizi A. Homayouni A. (2020). The effects of probiotics and prebiotics on mental disorders: a review on depression, anxiety, Alzheimer, and autism Spectrum disorders. Curr. Pharm. Biotechnol. 21, 555–565. doi: 10.2174/1389201021666200107113812, PMID: 31914909

[ref3] Bonaz B. Bazin T. Pellissier S. (2018). The Vagus nerve at the Interface of the microbiota-gut-brain Axis. Front. Neurosci. 12:49. doi: 10.3389/fnins.2018.00049, PMID: 29467611 PMC5808284

[ref4] Buie T. Fuchs G. J. 3rd Furuta G. T. Kooros K. Levy J. Lewis J. D. . (2010). Recommendations for evaluation and treatment of common gastrointestinal problems in children with ASDs. Pediatrics 125, S19–S29. doi: 10.1542/peds.2009-1878D, PMID: 20048084

[ref5] Chen Y. Xueying Z. Jiaqu C. Qiyi C. Huanlong Q. Ning L. . (2022). FTACMT study protocol: a multicentre, double-blind, randomised, placebo-controlled trial of faecal microbiota transplantation for autism spectrum disorder. BMJ Open 12:e051613. doi: 10.1136/bmjopen-2021-051613 PMC8804636 35105621

[ref6] Coretti L. Paparo L. Riccio M. P. Amato F. Cuomo M. Natale A. . (2018). Gut microbiota features in young children with autism Spectrum disorders. Front. Microbiol. 9:3146. doi: 10.3389/fmicb.2018.03146, PMID: 30619212 PMC6305749

[ref7] Cortese S. Wang F. Angriman M. Masi G. Bruni O. (2020). Sleep disorders in children and adolescents with autism Spectrum disorder: diagnosis, epidemiology, and management. CNS Drugs 34, 415–423. doi: 10.1007/s40263-020-00710-y, PMID: 32112261

[ref8] Cryan J. F. O’Riordan K. J. Cowan C. S. M. Sandhu K. V. Bastiaanssen T. F. S. Boehme M. . (2019). The microbiota-gut-brain Axis. Physiol. Rev. 99, 1877–2013. doi: 10.1152/physrev.00018.2018 31460832

[ref9] De Angelis M. Francavilla R. Piccolo M. De Giacomo A. Gobbetti M. (2015). Autism spectrum disorders and intestinal microbiota. Gut Microbes 6, 207–213. doi: 10.1080/19490976.2015.1035855, PMID: 25835343 PMC4616908

[ref10] Fattorusso A. Di Genova L. Dell’Isola G. B. Mencaroni E. Esposito S. (2019). Autism Spectrum disorders and the gut microbiota. Nutrients 11:11030521. doi: 10.3390/nu11030521, PMID: 30823414 PMC6471505

[ref11] Halkjaer S. I. Christensen A. H. Lo B. Z. S. Browne P. D. Gunther S. Hansen L. H. . (2018). Faecal microbiota transplantation alters gut microbiota in patients with irritable bowel syndrome: results from a randomised, double-blind placebo-controlled study. Gut 67, 2107–2115. doi: 10.1136/gutjnl-2018-316434, PMID: 29980607

[ref12] Han M. Yuan S. Zhang J. (2022). The interplay between sleep and gut microbiota. Brain Res. Bull. 180, 131–146. doi: 10.1016/j.brainresbull.2021.12.016, PMID: 35032622

[ref13] Iglesias-Vazquez L. Van Ginkel Riba G. Arija V. Canals J. (2020). Composition of gut microbiota in children with autism Spectrum disorder: a systematic review and Meta-analysis. Nutrients 12:12030792. doi: 10.3390/nu12030792, PMID: 32192218 PMC7146354

[ref14] Kang D. W. Adams J. B. Coleman D. M. Pollard E. L. Maldonado J. McDonough-Means S. . (2019). Long-term benefit of microbiota transfer therapy on autism symptoms and gut microbiota. Sci. Rep. 9:5821. doi: 10.1038/s41598-019-42183-0 30967657 PMC6456593

[ref15] Kang D. W. Adams J. B. Vargason T. Santiago M. Hahn J. Krajmalnik-Brown R. (2020). Distinct fecal and plasma metabolites in children with autism Spectrum disorders and their modulation after microbiota transfer therapy. mSphere 5:20. doi: 10.1128/mSphere.00314-20 PMC7580952 33087514

[ref16] Kong Q. Chen Q. Mao X. Wang G. Zhao J. Zhang H. . (2022). *Bifidobacterium longum* CCFM1077 ameliorated neurotransmitter disorder and Neuroinflammation closely linked to regulation in the kynurenine pathway of autistic-like rats. Nutrients 14:1615. doi: 10.3390/nu14081615, PMID: 35458177 PMC9031594

[ref17] Li N. Chen H. Cheng Y. Xu F. Ruan G. Ying S. . (2021). Fecal microbiota transplantation relieves gastrointestinal and autism symptoms by improving the gut microbiota in an open-label study. Front. Cell. Infect. Microbiol. 11:759435. doi: 10.3389/fcimb.2021.759435, PMID: 34737978 PMC8560686

[ref18] Li Y. Luo Z. Y. Hu Y. Y. Bi Y. W. Yang J. M. Zou W. J. . (2020). The gut microbiota regulates autism-like behavior by mediating vitamin B(6) homeostasis in EphB6-deficient mice. Microbiome 8:120. doi: 10.1186/s40168-020-00884-z, PMID: 32819434 PMC7441571

[ref19] Lin R. Liu W. Piao M. Zhu H. (2017). A review of the relationship between the gut microbiota and amino acid metabolism. Amino Acids 49, 2083–2090. doi: 10.1007/s00726-017-2493-3, PMID: 28932911

[ref20] Liu J. Gao Z. Liu C. Liu T. Gao J. Cai Y. . (2022). Alteration of gut microbiota: new strategy for treating autism Spectrum disorder. Front. Cell Dev. Biol. 10:792490. doi: 10.3389/fcell.2022.792490, PMID: 35309933 PMC8929512

[ref21] Liu Y. Sanderson D. Mian M. F. McVey Neufeld K. A. Forsythe P. (2021). Loss of vagal integrity disrupts immune components of the microbiota-gut-brain axis and inhibits the effect of *Lactobacillus rhamnosus* on behavior and the corticosterone stress response. Neuropharmacology 195:108682. doi: 10.1016/j.neuropharm.2021.108682, PMID: 34175326

[ref22] Martinez-Gonzalez A. E. Andreo-Martinez P. (2020). Prebiotics, probiotics and fecal microbiota transplantation in autism: a systematic review. Rev Psiquiatr Salud Ment (Engl Ed) 13, 150–164. doi: 10.1016/j.rpsm.2020.06.002, PMID: 32684346

[ref23] Montanari M. Martella G. Bonsi P. Meringolo M. (2022). Autism Spectrum disorder: focus on glutamatergic neurotransmission. Int. J. Mol. Sci. 23:3861. doi: 10.3390/ijms23073861, PMID: 35409220 PMC8998955

[ref24] Nankova B. B. Agarwal R. MacFabe D. F. La Gamma E. F. (2014). Enteric bacterial metabolites propionic and butyric acid modulate gene expression, including CREB-dependent catecholaminergic neurotransmission, in PC12 cells--possible relevance to autism spectrum disorders. PLoS One 9:e103740. doi: 10.1371/journal.pone.0103740, PMID: 25170769 PMC4149359

[ref25] Patusco R. Ziegler J. (2018). Role of probiotics in managing gastrointestinal dysfunction in children with autism Spectrum disorder: an update for practitioners. Adv. Nutr. 9, 637–650. doi: 10.1093/advances/nmy031, PMID: 30202938 PMC6140440

[ref26] Qi C. Ding M. Li S. Zhou Q. Li D. Yu R. . (2021). Sex-dependent modulation of immune development in mice by secretory IgA-coated *Lactobacillus reuteri* isolated from breast milk. J. Dairy Sci. 104, 3863–3875. doi: 10.3168/jds.2020-19437, PMID: 33612242

[ref27] Ristori M. V. Quagliariello A. Reddel S. Ianiro G. Vicari S. Gasbarrini A. . (2019). Autism, gastrointestinal symptoms and modulation of gut microbiota by nutritional interventions. Nutrients 11:2812. doi: 10.3390/nu11112812, PMID: 31752095 PMC6893818

[ref28] Rose D. R. Yang H. Serena G. Sturgeon C. Ma B. Careaga M. . (2018). Differential immune responses and microbiota profiles in children with autism spectrum disorders and co-morbid gastrointestinal symptoms. Brain Behav. Immun. 70, 354–368. doi: 10.1016/j.bbi.2018.03.025 29571898 PMC5953830

[ref29] Sharon G. Cruz N. J. Kang D. W. Gandal M. J. Wang B. Kim Y. M. . (2019). Human gut microbiota from autism Spectrum disorder promote behavioral symptoms in mice. Cells 177, 1600–1618.e17. doi: 10.1016/j.cell.2019.05.004, PMID: 31150625 PMC6993574

[ref30] Sun J. Qi C. Zhu H. Zhou Q. Xiao H. Le G. . (2019). IgA-targeted *Lactobacillus jensenii* modulated gut barrier and microbiota in high-fat diet-fed mice. Front. Microbiol. 10:1179. doi: 10.3389/fmicb.2019.01179, PMID: 31178854 PMC6542990

[ref31] Tan Q. Orsso C. E. Deehan E. C. Kung J. Y. Tun H. M. Wine E. . (2021). Probiotics, prebiotics, synbiotics, and fecal microbiota transplantation in the treatment of behavioral symptoms of autism spectrum disorder: a systematic review. Autism Res. 14, 1820–1836. doi: 10.1002/aur.2560, PMID: 34173726

[ref32] Vendrik K. E. W. Ooijevaar R. E. de Jong P. R. C. Laman J. D. van Oosten B. W. van Hilten J. J. . (2020). Fecal microbiota transplantation in neurological disorders. Front. Cell. Infect. Microbiol. 10:98. doi: 10.3389/fcimb.2020.00098, PMID: 32266160 PMC7105733

[ref33] Wang Y. Li N. Yang J. J. Zhao D. M. Chen B. Zhang G. Q. . (2020). Probiotics and fructo-oligosaccharide intervention modulate the microbiota-gut brain axis to improve autism spectrum reducing also the hyper-serotonergic state and the dopamine metabolism disorder. Pharmacol. Res. 157:104784. doi: 10.1016/j.phrs.2020.104784, PMID: 32305492

[ref34] Xu H. M. Huang H. L. Zhou Y. L. Zhao H. L. Xu J. Shou D. W. . (2021). Fecal microbiota transplantation: a new therapeutic attempt from the gut to the brain. Gastroenterol. Res. Pract. 2021, 6699268–6699220. doi: 10.1155/2021/6699268, PMID: 33510784 PMC7826222

[ref35] Zhang F. Luo W. Shi Y. Fan Z. Ji G. (2012). Should we standardize the 1,700-year-old fecal microbiota transplantation? Am. J. Gastroenterol. 107, 1755–1756. doi: 10.1038/ajg.2012.251 23160295

[ref36] Zhao H. Gao X. Xi L. Shi Y. Peng L. Wang C. . (2019). Mo1667 FECAL MICROBIOTA TRANSPLANTATION FOR CHILDREN WITH AUTISM SPECTRUM DISORDER. Gastrointest. Endosc. 89:AB512-AB513. doi: 10.1016/j.gie.2019.03.857

[ref37] Zou R. Xu F. Wang Y. Duan M. Guo M. Zhang Q. . (2020). Changes in the gut microbiota of children with autism Spectrum disorder. Autism Res. 13, 1614–1625. doi: 10.1002/aur.2358 32830918

